# Comparative Bioavailability of Synthetic B12 and Dietary Vitamin B12 Present in Cow and Buffalo Milk: A Prospective Study in Lactovegetarian Indians

**DOI:** 10.3390/nu11020304

**Published:** 2019-02-01

**Authors:** Namita Mahalle, Vijayshri Bhide, Eva Greibe, Christian W. Heegaard, Ebba Nexo, Sergey N. Fedosov, Sadanand Naik

**Affiliations:** 1 Department of Pathology, Deenanath Mangeshkar Hospital and Research Center, Pune 411004, India; pnmahalle@gmail.com (N.M.); drvijayshrimb@yahoo.com (V.B.); 2 Department of Clinical Biochemistry and Institute of Clinical Medicine, Aarhus University Hospital, DK-8200 Aarhus N, Denmark; evagreibe@gmail.com (E.G.); ebbanexo@rm.dk (E.N.); 3 Department of Molecular Biology and Genetics, Aarhus University, DK-8000 Aarhus C, Denmark; cwh@mbg.au.dk

**Keywords:** vitamin B12, cobalamin, cyano-B12, hydroxo-B12, B12 supplementation, B12 deficiency, milk, cow, buffalo

## Abstract

We assessed improvements in the vitamin B12 status of Indian lactovegetarians receiving four weeks supplementation with natural B12 in milk versus cyano-B12 in capsules. Three groups (*n* = 22, 23, 22) received daily oral doses of cyano-B12 (2 × 0.76 µg) or milk (2 × 200 mL) from a cow or buffalo (amounting to B12 ≈ 2 × 0.76 µg). Their blood was examined at baseline and each following week. The baselines (median (min/max)) indicated a low B12 status: plasma B12 (116(51/314)) pmol/L, holotranscobalamin (holoTC) (30(7/119)) pmol/L, total homocysteine (Hcy) (24(10/118)) µmol/L, methylmalonic acid (MMA) (0.58(0.15/2.2)) µmol/L and combined B12 index (cB12) (−1.32 − (−3.12/+0.29)). Shifts from the baselines (B12, holoTC, cB12) and ratios to the baselines (Hcy, MMA) were analyzed over time. The cyano-B12 treatment gave more total B12 in plasma at week one (+29 pmol/L, *p* = 0.004) but showed no further increase. Other biomarkers changed more comparably between the three groups (*p* ≥ 0.05): holoTC showed a transient spike that leveled off, Hcy finally decreased to 0.8 × baseline, while MMA showed marginal changes. The combined indexes improved comparably (*p* = 0.6) in all groups (+0.2(−0.3/+0.9), *p* ≤ 0.002). In conclusion, the tested formulations similarly improved B12 status, but did not normalize it.

## 1. Introduction

Vitamin B12 (B12, cobalamin) is an essential water-soluble molecule, primarily present in foods of animal origin [1]. B12 deficiency is characterized by a decrease in total plasma B12, including the plasma B12 bound to its transport protein transcobalamin (holotranscobalamin, holoTC), which signifies the fraction of total plasma B12 readily available for tissue uptake [1]. B12 deficiency results in increased concentrations of methylmalonic acid (MMA) and homocysteine (Hcy), due to decreased activity of methylmalonyl-coenzyme A mutase and methionine synthase, respectively [1,2,3,4,5]. While severe deficiency can cause megaloblastic anemia and permanent neurological damage, early physiological manifestations are generally subtle [1,6].

Retrospective assessments of dietary rations have shown that an improvement in B12 status highly correlates with ingestion of dairy products, whereas its relations to intake of meat, poultry, eggs, or fish are weaker [7]. Intervention studies have also revealed that the B12 status of vegetarians is positively associated with their intake of dairy products [8], suggesting that milk and milk products are efficient carriers of highly bioavailable B12. Cow and buffalo milk contain approximately the same amount of B12, 2–7 µg/L [9]. While cow milk is the dominant milk form consumed in Western countries, buffalo milk is widely consumed in India. The form of B12 present in both types of milk is mainly hydroxo-B12 (HO-B12) [10], which is different from cyano-B12 (CN-B12), a synthetic form of the vitamin typically present in vitamin pills and food fortification.

In humans, oral uptakes of CN-B12 and HO-B12 are similar [11]. Employing an animal model, we have previously demonstrated that, though the two forms of B12 are absorbed alike, they distribute differently within the body [12,13]. Thus, CN-B12 had a tendency to accumulate in the blood and the kidneys, whereas HO-B12 targeted mainly to the liver. These findings question whether the two vitamin forms are of equal value for improvement of B12 status.

The present study was undertaken to compare biomarkers of B12 status during four weeks of supplementation with equivalent amounts of B12, administered in either vitamin capsules (CN-B12), cow milk (dietary HO-B12), or buffalo milk (dietary HO-B12), in Indian individuals with a low B12 status.

## 2. Materials and Methods

### 2.1. Study Design and Supplementation Products

**Figure 1:**
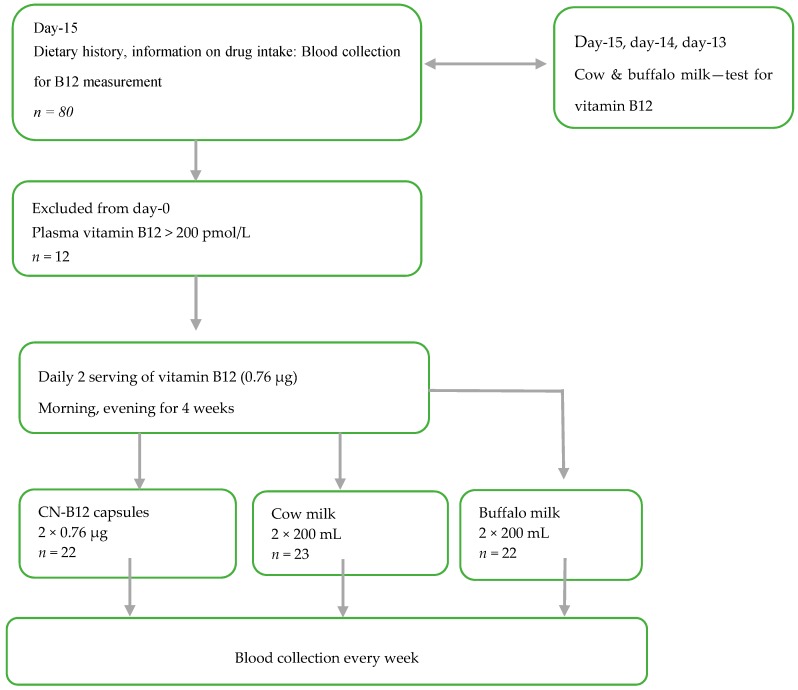
Scheme of the study.

This randomized intervention trial (Figure 1 and Supplementary Table S1) was designed to investigate the B12 status in lactovegetarian Indian individuals, supplemented for four weeks with two doses of B12 (morning and evening), as either vitamin capsules with CN-B12 = 0.76 µg (CN group) 200 mL of cow milk (cow milk group), or 200 mL of buffalo milk (buffalo milk group), with both milk types containing B12 ≈ 0.78 µg in 200 mL (see measurements in Supplementary Table S2, as well as further details in ref. [8]). CN-B12 capsules were prepared in Aarhus, Denmark as previously described [14,15]. The compliance of the milk/capsule intake was monitored by the Whatsapp online program. Each participant was contacted to confirm capsule/milk intake every day, in the morning and evening, through Whatsapp. The whole exercise was free of cost. Deenanath Mangeshkar Hospital and M/S Chitale Bandhu dairy firm entered into a memorandum of agreement stating that Chitale Bandhu would deliver (2 × 200 mL × 25) cow milk for 25 participants and 2 × 200 mL × 25) of buffalo milk for another 25 participants) to Deenanath Mangeshkar Hospital and Research Center, Pune, India. Fifty milk packets each were delivered every morning. Each participant was given two packets to be consumed, one at night and the other next day morning. They poured the milk in a vessel and heated it until it was boiling and then cooled immediately and drank. This procedure continued for 4 weeks.

### 2.2. Participants

Participants (*n =* 68; 30 males and 38 females) between the age of 18–50 years were recruited from the Pune area in India, and the study was carried out at Deenanath Mangeshkar Hospital and Research Center, Pune, India, from April 2017 to June 2017. Inclusion criterion was a lactovegetarian diet. Exclusion criteria were the use of vitamin pills containing > 1 µg B12 within the last two weeks, drugs (methotrexate, antacids, and metformin) known to influence B12 absorption/biomarkers, and known chronic systemic disease. Based on our knowledge from a previous B12 intervention study in a comparable Indian population [15], the number of participants included in each intervention group was calculations using a statistical power of 80% and a two-sided significance level of 5%. Anticipating a mean plasma B12 increase of 20% for the participants receiving intervention with two daily doses of 0.76 µg CN-B12 for four weeks, the sample size calculations suggested *n* = 19 in each group. In order to account for possible dropouts, 68 participants were divided evenly and randomly into three groups: capsule group, cow milk group, and buffalo milk group (1 female excluded post-trial due to a baseline value for holoTC above the upper limit for the reference interval), see result section. The participants were allowed to continue on their regular lactovegetarian diet throughout the study. The participants received no compensation for participation in the study.

Folate and vitamin B12 deficiency were defined as plasma concentrations <4.54 nmol/L and <150 pmol/L, respectively [8]. Anemia was defined as a hemoglobin concentration <7.5 nmol/L (120 g/L) in females and <8.0 nmol/L (130 g/L) in males [8]. Macrocytosis was stipulated as MCV >100 fl [8].

The investigation was performed within the confines of the Helsinki Declaration II, and the study was approved by the Institutional Ethics Committee of Deenanath Mangeshkar Hospital and Research Center (project No. 2015_APR_SN_167). All individuals gave their informed consent before inclusion in the study. The study is registered with Clinical Trial Registry of India, bearing the indicator CTRI/2017/08/009342.

### 2.3. Blood Sampling and Biochemical Methods

At baseline and each week throughout the study, non-fasting blood samples were drawn into EDTA and plain vacutainers and kept in an ice-cold box. Hemoglobin and mean cell volume were determined on the XN 3000 Hematology Analyzer (Sysmex, Japan) [8]. Blood samples were centrifuged (10 min at 2300 g) within two hours after withdrawal and serum/plasma was stored at −56 °C until analysis/shipment of samples. Plasma creatinine (only baseline), cholesterol, triglycerides, and HDL-cholesterol were measured in the samples collected at the baseline and at the end of the study (4 weeks) using ISO (International Organization for Statndardization) certified enzymatic methods on the RX Imola (Randox Laboratories, London, UK) [8]. Plasma B12 (cobalamin, B12), holotranscobalamin (holoTC), folate, and total homocysteine (Hcy) concentrations were measured using ARCHITECT (Abbott, Columbus, OH, USA) every week in batches, each batch having internal quality control samples [8]. Plasma aliquots were shipped to Aarhus University Hospital, Aarhus, Denmark, on dry ice for analysis of MMA, measured in one run for all participants. MMA was quantified by liquid chromatography-tandem mass spectrometry on the AB SCIEX Tripel Quad 5500 System (AB SCIEX, IL, USA) [15]. Intra-batch coefficients of variation (in percent) for vitamin B12, holoTC, Hcy, folate and MMA were 4.5%, 6.3%, 4.2%, 5.2% and 5.4% and inter-batch coefficients were 4.8%, 6.9%, 4.5% and 5.8%, respectively.

### 2.4. Combined Analysis of Markers

The four-component combined indicator of B12 status 4cB12 (or its three-component variant 3cB12 at occasional missed analysis, or 2cB12 at separate analysis of B12-related vs metabolite-related markers), was calculated from the measurements of serum B12, holoTC, MMA, and Hcy using the formula presented by Fedosov et al. [16]. The effect of plasma folate on the combined B12 index was examined by entering its values into the calculation spreadsheets [16].

### 2.5. Statistical Procedures and Software

Examination of the overall identity of baselines between the supplementation groups (for each given marker) was conducted by a non-parametric Kruskal-Wallis test. The pairwise comparisons between the groups were performed by a Steel-Dwass test (multiple comparisons without assumption of normality). The final outcome of the treatment (baseline vs. endpoint) was assessed by a Wilcoxon signed rank test for paired groups (without assumption of normality) [17]. The shift of scaled responses (Section 2.6) from the baseline at each time point was assessed by the parametric paired *t*-test (to maintain consistency with the parametric method of least squares also used in these charts). The overall identity of all scaled responses at each time point was examined by single factor ANOVA, while assessment of their pairwise identity was done by a Tukey-Kramer test (with correction for multiple comparisons).

Statistical analysis and fitting of the data (Section 2.7) employed KyPlot 5.0 software (free software from Kyenslab Inc, Tokyo, Japan).

### 2.6. Correction for Baselines and Smoothing of Data

All responses were corrected for the baselines. Time-dependent changes (Δ*y*) in plasma B12 and holoTC (*y*) from their respective baselines (*y*_0_) were calculated for each participant as Δ*y* = *y* − *y*_0_. This scaling was expected to suppress the excessive dispersion caused by different starting points. Changes in MMA and Hcy over time were presented as ratios (R(*y*) = *y*/*y*_0_) between the concentration at a given time point (*y*) and the concentration at the baseline (*y*_0_). Such normalization partially compensated for larger (smaller) responses at higher (lower) initial metabolites, usually observed at identical changes in the B12 statuses, see also ref. [15].

All adjusted responses were plotted over time and smoothed. The latter procedure was intended to compensate for a relatively high noise to signal ratio and expose the shape of dependency to simplify selection of the fitting equation. Smoothing was done individually for each participant and involved a two-dimensional averaging of two adjacent points (for example, for weeks 1 and 2) rendered as Δ*y*_1,2_ = (Δ*y*_week 1_ + Δ*y*_week 2_)/2 on the marker axis and as *x*_1,2_ = (1 + 2)/2 = 1.5 (i.e., week 1.5) on the time axis. As a result, the original data at weeks 1, 2, 3, and 4 were transformed to their average values at weeks 1.5, 2.5, and 3.5, respectively.

### 2.7. Approximating Models and Comparison of Supplements

The response of a marker over time was approximated by an arbitrary function (from the set of equations below), capable of reproducing the shape of dependency, but claiming no physiological meaning:

*y* = *P*_1_ + *P*_2_ · *x* + *P*_3_ · *x*^2^ (1)

*y* = *P*_1_ + *P*_2_ · *x* + *P*_3_ · (*x +* 10^−20^)^0.5^ (2)

*y* = *P*_1_ + *P*_2_ · (1 − *e*^−*P*^^3^^· *x*^) (3)

Here *y* stands for the relevant scaled marker (Δ*y* or R(*y*)); *P*_1_ is a baseline value of *y* (fixed at 0 or 1, depending on the context); *P*_2_ and *P*_3_ represent the floating parameters assessed by fitting; *x* is the time of treatment. The value of *x* received a negligible increment of 10^−20^ in Equation (2) to avoid *x* = 0, forbidden in a power function. The equations were chosen to guarantee a reasonably accurate shape of the curve, while avoiding “superfluous” floating parameters, frequently tending to infinity and/or exhibiting a very high likelihood for error. The shapes of the functions obeyed: (i) a linear start followed by a bending for Equation (1); (ii) an initial bending followed by a linear continuation for Equation (2); (iii) a quasi-linear start tending to a plateau for Equation (3). Equations (1, 2, or 3) were applied in accordance with the detected shapes of dependencies.

Only one function (e.g., Equation (2) was used to approximate the datasets for each given marker (e.g., ΔB12) to simplify the parametric comparison of fits. Parameters of this fitting function (e.g., *P*_1_, *P*_2_, *P*_3_) and their errors (e.g., SE_1_, SE_2_, SE_3_) for each supplementation were estimated by the method of least squares. The “baseline parameter” *P*_1_ was fixed at either 0 (for ΔB12, ΔholoTC) or 1 (for R(MMA), R(Hcy)). The fixed coefficient *P*_1_ was retained in the equation (as well as in the covariance table) to account for its error, affecting the two floating parameters *P*_2_ and *P*_3_.

Presence or absence of a response was assessed by *t*-test, comparing each fitting parameter to its reference value (0 or 1 depending on the context). The probability (*p*) of a “zero function” (with all parameters equal to their reference values, 0 or 1) was calculated as the probability of several simultaneous events, e.g., *p* = *p*_1_·*p*_2_·*p*_3_, with *p*_1_ = 1 for the fixed parameter *P*_1_.

The same approach was used to assess, whether the two supplementations (e.g., “A” and “B”) gave the identical time-dependent curves (e.g., “curve A” = “curve B”). The respective sets of fitting parameters (from the same function) were examined on their equality in pairs (*P*_1A_ = *P*_1B_, *P*_2A_ = *P*_2B_, *P*_3A_ = *P*_3B_) by *t*-test, essentially as discussed in ref [18], with *t*_iAB_ = (*P*_iA_ − *P*_iB_)/*SE*_iAB_, *SE*_iAB_^2^ = *SE*_iA_^2^ + *SE*_iB_^2^ and degrees of freedom *df* = *n*_A_ + *n*_B_ − 2*m* (*n* = total number of points in sets “A” and “B”, *m* = the number of parameters, i.e., 3 in the current case). The probabilities of identity (*p*_1_, *p*_2_, *p*_3_) for the individual parameter pairs were calculated, finally giving the overall identity between the two curves (*p* = *p*_1_·*p*_2_·*p*_3_). Multiple (three) comparisons (“A” = “B”, “B” = “C” and “A” = “C”) increased the likelihood of a “false discovery” (type I error), and this issue was addressed by applying a Holm-Bonferroni correction to the critical *p*-values, changed from e.g., *p* = 0.05 to 0.05/3 (for the lowest *p* from three comparisons), followed by 0.05/2 and 0.05/1 (for the sequentially higher *p*).

## 3. Results

### 3.1. Characteristics of Participants and Supplements

Sixty-eight lactovegetarians (30 males and 38 females) were randomly assigned to one of three groups for a four week intervention period (Table 1). One female individual was excluded (post hoc) from the buffalo group due to baseline plasma holoTC above the standard reference interval (Table 1). The participants had no recognized chronic systemic diseases, clinical symptoms of B12 deficiency, and were not taking B12 supplementation or any drugs known to influence B12 absorption. None of the participants was clearly anemic with hemoglobin of (mean(min/max)) = (8.1(7.8/8.5)) mmol/L.

Eleven subjects had baseline B12 levels above the lower limit of the reference interval (200 pmol/L), but the overall cohort median (110 pmol/L, *n =* 67) was below the reference interval. Low B12 status was further supported by overall low holoTC and elevated concentrations of MMA and tHcy (Table 1). The combined indicators of B12 status (4cB12: total B12, holoTC, MMA, tHcy) showed a median value of −1.32, indicating a “low B12 status” [16]. Plasma folate and its effect on the combined indicators were also examined at baseline (Table 1). It appeared that correction for folate increased cB12 by Δ = 0.06 (mean) and 0.13 (median), which had no bearing on the interpretation of results, as the cB12 gradation covered much broader spans of e.g., +1 ± 0.5 and 0 ± 0.5 (both adequate), −1 ± 0.5 (low B12), −2 ± 0.5 (possibly deficient) etc. [16]. Further assessments of folate were not undertaken.

The overall design of the study is depicted in Figure 1.

The groups received either two daily doses of B12 in a capsule form (1 capsule with CN-B12 = 0.76 µg), or with the same regularity, two servings of cow or buffalo milk (200 mL, B12 ≈ 0.78 µg), see scheme in Figure 1 and Supplementary Tables S1 and S2. Cow and buffalo milk were analyzed for their contents of endogenous B12 [8] before and during the intervention (*n* = 5 for each) on days −15, −14, −13, day 0 and day+15. No significant difference between the samples from the two species was found (Supplementary Table S2).

**Figure 2:**
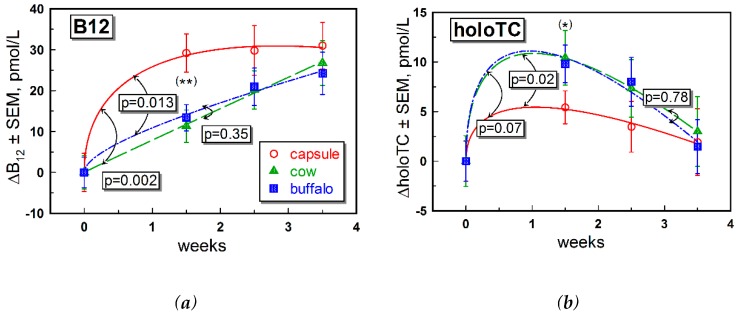
Time-dependent changes of the B12 (**a**) and holoTC (**b**) in the course of the three supplementation methods notated as “capsule”, “cow”, and “buffalo”. Both (**a**) and (**b**) present the data as mean values ± SEM for the supplementation with CN-B12 capsules (○, red); cow milk (▲, green); and buffalo milk (■, blue). The solid lines of respective colors show the best approximations by Equation (2). Double arrows (↕ black) indicate the probability of identical fitting parameters in pairs cow = buffalo, capsule = cow and capsule = buffalo. The significance levels of *p* < 0.05/3, 0.05/2 and 0.05/1 for three comparisons were accepted for the lowest to the highest *p*-values observed (Holm-Bonferroni correction, see methods Section 2.7). Symbols (*) and (**) indicate differences (ANOVA, *p* < 0.05 and 0.01, respectively) between the datasets at the particular time points. HoloTC: holotranscobalamin.

**Figure 3:**
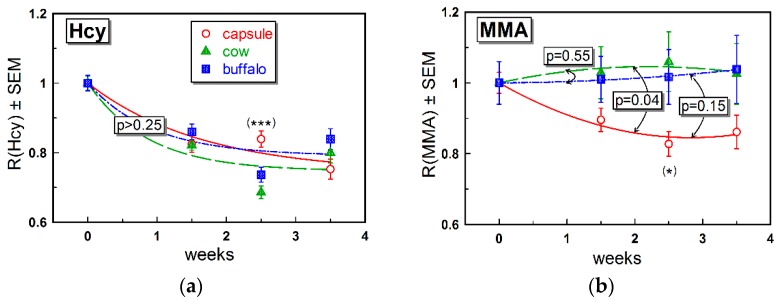
Time-dependent changes in (**a**) Hcy (fitted by Equation (3)) and (**b**) MMA (fitted by Equation (1)), both normalized to the respective baselines. Other notations and descriptions of the three groups are as in the legend to Figure 2. Symbols (*) and (***) indicate differences (ANOVA, *p* < 0.05 and 0.001, respectively) between the datasets at the particular time points.

All three supplementation groups had comparable baseline values of plasma B12, MMA, combined B12 markers, folate, hemoglobin, creatinine and lipids (Table 1). Some difference between the groups was detected for Hcy, which was elevated in the capsule vs. cow and buffalo milk groups (Table 1). Plasma holoTC concentrations were similar in the capsule and buffalo milk groups, but higher in the cow milk group (Table 1). These differences in the starting values were counteracted by subtraction of the baselines for total B12 and holoTC, and division by the baseline for MMA and Hcy, when reporting the changes observed upon the interventions in Figure 2 (Figure 3, see Section 2.6 for further details).

### 3.2. Time Dependent Changes in the Biomarkers of B12 Status

Shifts of total plasma B12 over time are depicted in Figure 2a, where we found that all mean points lay above the baseline already at week 1.5 (*p* ≤ 0.001). The sets of points from the three supplementations differed from each other at week 1.5 (*p* = 0.004), with the capsule group ranked highest. At other time points (weeks 2.5 and 3.5), the points considerably overlapped (*p* > 0.35). The shapes of the fitting curves revealed a difference in responses between capsule supplementation on the one hand, and the milk variants on the other hand. Supplementation with CN-B12 capsules caused a steep increase in ΔB12, whereas ingestion of milk caused a steady and nearly linear increase over four weeks, irrespective of milk source. Parameters of the fitting Equation (2) (Supplementary Table S3) provided a quantitative confirmation that the capsule curve differentiated itself from the records for both cow milk (*p* = 0.002 < Holm-Bonferroni critical value of 0.05/3) and buffalo milk (*p* = 0.013 < 0.05/2). In contrast, the two milk curves had a high mutual identity (*p* = 0.35 > 0.05/1).

The records of ΔholoTC (Figure 2b) showed a temporary “spike” around week 1.5, visible in all groups (*p* < 0.004 at week 1.5). This spike leveled off at week 3.5, tending to zero (*p* > 0.41). The data at each individual time showed a general overlap for all three supplementations, though with some indication of a difference at week 1.5 (*p* = 0.05), where the capsule group was ranked lowest. The general fits by Equation (2) (see results in Supplementary Table S3) pointed to a reasonably high similarity of all three curves in Figure 2b: capsule = buffalo (*p* = 0.02 > 0.05/3), capsule = cow (*p* = 0.07 > 0.05/2) and cow = buffalo (*p* = 0.78 > 0.05/1), employing critical *p*-values of Holm-Bonferroni.

The changes in the R(Hcy) = Hcy/Hcy_0_ ratio revealed a nearly uniform decrease in this marker, irrespective of the supplementation method (Figure 3a). At each time point, the ratio differed from the baseline (*p* < 0.001), with a considerable overlap between the supplementation groups, except for week 2.5, where the result for capsule treatment seemingly deviated upwards from the two milk groups (*p* < 0.001). This deviation was not confirmed by general fitting analysis (Equation (3) and Supplementary Table S3), and a high mutual identity of all approximating curves was detected (*p* > 0.25).

The shift of the R(MMA) = MMA/MMA_0_ ratio from its baseline (Figure 3b) was significant only for the capsule group (*p* < 0.005 for all time points starting from week 1.5). However, a comparison between the groups revealed only a marginal difference at week 2.5 (*p* = 0.05), where the points for capsule supplementation deviated downward from the points for milk supplementations. A more detailed inspection of the data in milk groups revealed the presence of 2–3 “opposite responders”, with R(MMA) considerably deviating upwards from the baseline (Supplementary Figure S1). Yet, the bulk of points was similar to the capsule treatment. A general approximation of the three datasets by Equation (1) (see results in Supplementary Table S3) confirmed a response to capsule ingestion (*p* = 0.002), whereas both milk groups exhibited a “zero function” (*p* > 0.46). The fitting analysis showed, at the same time, a similarity among the three supplementation curves, all giving a mutual identity above the critical *p*-values: capsule = cow (*p* = 0.04 > 0.05/3), capsule = buffalo (*p* = 0.15 > 0.05/2) and cow = buffalo (*p* = 0.55 > 0.05). Taking into account all facts above, we selected a conservative interpretation, claiming a reasonably high similarity and a low response in R(MMA) for all three supplementation methods.

### 3.3. Analysis of Endpoint Measurements

Table 2 presents the endpoint results (the marker concentrations at 3.5 weeks of supplementation, medians, and range). A paired test of identity between the data at week 3.5 and the respective baselines was performed for each supplementation method. This analysis resembled the previous assessment of endpoints in Figure 2 and Figure 3, with a few exceptions. Thus, Table 2 uses a nonparametric test, whereas Figure 2 and Figure 3 present the *t*-tests with the assumption of normality (to be consistent with the parametric fitting procedure in these figures). In addition, Figure 3 depicts the normalized responses (related to baseline values), whereas Table 2 shows comparisons without such scaling. The endpoints of all supplementations indicated clear changes in total B12, Hcy, and the combined index cB12. Changes in MMA were expressed to a lower degree (and only for capsule supplementation at the best). The final levels of holoTC coincided with starting levels.

### 3.4. Combined Analysis

The responses to supplementation were interpreted in terms of the combined indicator of B12 status (cB12), which included either all four markers or two markers (2cB12): either B12 and holoTC or Hcy and MMA. We intended to assess (i) the overall average response of four markers (4cB12) to one or another intervention, and (ii) the degree of synchronization between the changes in the “pure B12 index” (2cB12 for B12 & holoTC) and the “metabolic index” (2cB12 for Hcy & MMA). It should be noted that all variants of cB12 were scaled to the same span of units, and therefore a steady-state shift in all B12 indexes was expected to be identical irrespective of the initial B12 status.

**Figure 4:**
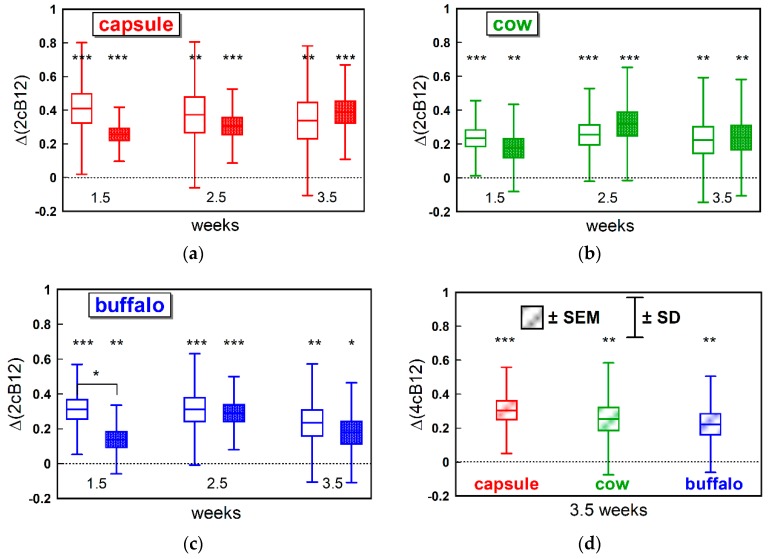
Changes in the combined indexes of B12 status during supplementations with (**a**) CN-B12 capsules (*n* = 17–22, depending on the number of missed measurements); (**b**) cow milk (*n* = 21–23); (**c**) buffalo milk (*n* = 19–22). Panels **a**, **b,** and **c** present shifts from the baselines in the two-component indexes of B12 status (2cB12), calculated from either the B12-related markers (total B12 and holoTC, open boxes) or the metabolite-related markers (total Hcy and MMA, closed boxes). (**d**) Comparison of all three supplementation groups at the endpoint of intervention (average of weeks 3 and 4, see Section 2.6). The panel presents changes in the 4cB12 (covering all four markers). Notation in all panels follows the same pattern: vertical boxes with centerlines indicate means ± SEM; vertical whiskers show the span of mean ± SD; stars (***, **, and *) notate the probabilities (*p* ≤ 0.001, ≤ 0.01, and ≤ 0.05) of zero shifts from the baseline (horizontal dashed line). The horizontal brace in panel **c** indicates a difference in the indexes for 2cB12 (B12 + holoTC) and 2cB12 (MMA + Hcy) at week 1.5.

The data in Figure 4 show significant positive shifts Δ(2cB12) for both the “pure B12 index” and “metabolic index” for all supplementations: CN-B12 capsules (Figure 4a), cow milk (Figure 4b), and buffalo milk (Figure 4c). At the endpoint, the shifts in 4cB12 (covering all four markers) were also statistically significant (Figure 4d): +0.30 (CN-group, *p* < 0.001), +0.25 (cow milk-group, *p* = 0.001) and +0.22 (buffalo milk-group, *p* = 0.002). The three responses did not differ (*p* = 0.49, ANOVA).

The changes in the “partial indexes” Δ(2cB12) for B12+holoTC and Hcy + MMA in Figure 4a–c were aligned against each other at each time point. Somewhat smaller metabolic responses vs. B12-related responses were found at week 1.5 in all supplementation groups, and this difference was statistically significant in the buffalo milk group (*p* = 0.02). A delay in metabolic response at week 1.5 was more evident in a pooled dataset (including all three groups, not shown), where the “metabolic index” was smaller than “B12 index” by mean ± SEM = −0.125 ± 0.045 (*p* = 0.007). No difference was found for the weeks 2.5 and 3.5 (*p* > 0.74).

## 4. Discussion

We reported changes in biomarkers related to vitamin B12 status in a lactovegetarian Indian population during four weeks of daily supplementation with ≈ 1.5 µg of B12, administered as two oral doses of CN-B12 (vitamin capsules) or 2 × 200 mL of cow or buffalo milk, containing the natural forms of B12 (mainly HO-B12).

A weakness of this study concerns differences in baselines for holoTC and Hcy among the three intervention groups, despite similar levels of the circulating total B12 (Table 1). The dispersion of baselines within each supplementation group was also high, indicating that the selection criterion of total plasma B12 < 200 pM was not sufficiently strict. We attempted to counteract the variations of baselines by recording the difference between actual and baseline values (for total B12 and holoTC) or the ratio between actual and baseline values (for Hcy and MMA). Another weakness was the lack of information on dietary intake during the study period. Theoretically, groups offered supplementation with milk could have decreased the intake of other food items, thereby possibly underestimating the benefit of the milk intervention.

The recommended daily dose of B12 is 2.4 µg, based on Western standards [19], or 1 µg, based on Indian standards [20]. It has been estimated that the daily intake by Indian lactovegetarians is at the magnitude of B12 ≈ 1.65 µg/day [8], implying a proportionally lower total body store of B12, in comparison to a Western population with Σ B12 ≈ 3 mg [19] and references thereof). An increment of ≈1.5 µg/day increased the intake of B12 by our cohort to a dose slightly exceeding the Western recommended allowance [19]. Thereby we addressed the issue of whether such a dose can improve the biochemical markers of B12 status within a four week trial, though the complete replenishment of B12 body stores was out of reach after the overall intake of Σ B12 ≈ 90 µg. We also anticipated that any major benefit of milk supplementation, as compared to CN-B12 capsules, would be more transparent at low doses (i.e., not saturating the transport system). For convenience, the milk servings were administrated in the mornings and evenings. The same pattern was followed with the vitamin capsules, even though they are generally ingested only once a day. Our choice was also driven by the fact that more B12 is believed to be absorbed from a divided dose than from a single dose [21].

Supplementation with CN-B12 capsules or one or the other form of milk provided small but detectable differences “within the group” in most of the B12 biomarkers. Differences between the groups were also observed in a few instances (e.g., for changes in total B12, Figure 2a). Differences between the groups can be ascribed to (i) the particular form of B12 administered (synthetic CN-B12 in capsules or the natural vitamin forms (e.g., HO-B12) in milk), and/or (ii) the matrix of the food supplements.

It should be taken into account that all pasteurized milk (cow and buffalo) in India is boiled before ingestion. Accordingly, a considerable portion of B12 is released from the endogenous milk carriers, haptocorrin (HC), and/or transcobalamin (TC). This paves the way for B12 binding to casein, the main protein fraction of ruminant milk, which, besides being heat stable, also has the capacity to bind large amounts of HO-B12 [22]. Therefore, a possible matrix effect is likely to be similar for both types of milk, irrespective of their specific binders, generally destroyed by boiling. Intake of milk, as well as other food items, stimulates both gastric acid production and the secretion of intrinsic factor [1,23,24]. The combination of these effects is expected to promote a more efficient uptake of B12 (in comparison to a vitamin capsule ingested without any bulk filling). Casein has long been known to stretch the time of intestinal transit, thereby increasing the duration of B12 uptake and reducing the saturation degree of its absorption system. Finally, some effects of bioactive peptides in milk may be at play.

When interpreting our results concerning the increase in total B12 and holoTC, the dynamics of plasma B12 also has to be considered. B12 in blood circulates in two pools: one is bound to heptocorrin (HC) and the other to transcobalamin (TC). HC is almost fully saturated with B12 (at least in B12 replete individuals), it has a slow turnover rate and shows marginal importance for B12 tissue delivery [1,24]. TC circulates mainly unsaturated, it has a fast turnover rate and plays a major role in B12 tissue delivery [1,24]. Newly absorbed B12 preferentially binds to the surplus TC, producing a TC-B12 complex (holoTC) taken up by the cells. The excessive or unprocessed cellular B12 is released back to the blood with a repetition of the aforementioned cycles, leading to a gradual accumulation of the unprocessed vitamin within the slow-exchanging HC pool. All in all, the total plasma B12 can be interpreted as the “inert” HC complex (carrying most of the circulating B12), whereas holoTC reflects the fast-exchanging “active” component of the vitamin pool, that in a blood sample usually accounts for 1/8–1/4 of the total B12 content [25].

We observed higher levels of total B12 after ingestion of CN-B12, compared to ingestion of the natural B12 forms (mainly HO-B12) present in milk, which was in accord with our previous data for supplementation of CN-B12 vs. HO-B12 in capsules [13,15]. The increase in total B12 after administration of CN-B12 was almost exclusively driven by accumulation of HC-bound B12, as follows from a high proportion of total ΔB12: ΔholoTC ≈ 6–10 (Figure 2a vs. Figure 2b, ○-red symbols). In contrast, a much lower accumulation of B12 on HC was observed after administration of milk (Figure 2a vs. Figure 2b, ▲-green and ■-blue symbols). We hypothesize that a high level of total B12 in the blood after administration of CN-B12 (in comparison to HO-B12 from milk) originated from accumulation of CN-B12 on HC, because more processing cycles are seemingly required to remove the protective CN-group in CN-B12. The turnover rates of holoTC were apparently similar for all supplementations, with a marginally better response in the milk-groups (Figure 2b). Comparable changes in holoTC were observed in our earlier works upon administration of CN-B12 vs. HO-B12, where an insignificantly higher increase was observed for the CN-B12 supplementation [13,15].

The changes in the metabolic markers MMA and Hcy were rather small (Figure 3 and Table 2). As discussed above, a low impact on metabolites may reflect a marginal alteration in the body B12 store and thereby cellular content of B12. The effect on Hcy (Figure 3a) was more pronounced and similar in all groups (Figure 3b, see also Table 2). Regarding MMA, we assumed comparably low responses irrespective of supplementation. A more explicit change in Hcy was in accord with our previous data, indicating Hcy as the metabolic marker responding first to an increased intake of B12 [8].

We used the newly introduced combined indicator of B12 status (cB12) to compare the three supplementations and assess synchronization between the “pure B12” index (including B12 and holoTC) and the “metabolic index” (including MMA and Hcy). Regardless of supplementation, the “metabolic index” showed a lower response at week 1.5. We believe this represents the fact that B12 and holoTC have to increase in plasma prior to entering the cells and benefiting intracellular B12 metabolism. The observation is in agreement with our previous findings indicating that a daily oral supplementation of 3-µg CN-B12 or 3-µg HO-B12 hardly changes the metabolic markers MMA and Hcy in lactovegetarian Indians [13,15]. Uniting all our measures into the 4cB12 index by the end of the study, we found comparable values for the three intervention groups, suggesting that CN-B12, cow milk, and buffalo milk have comparable effects on the measured markers of B12 status in the Indian cohort. This effect was judged to be by far insufficient to reach a fully replete B12 status that would require a 4cB12 value of 0 ± 0.5 or more [16].

## 5. Conclusions

We reported that two servings of vitamin capsules with CN-B12, cow milk, or buffalo milk (containing mostly HO-B12) are equally efficient in improving biomarkers of B12 deficiency. Our observation that the capsules give a higher increase in the total plasma B12, does not signify a potential benefit of CN-B12, but reflects accumulation of B12 on the inert protein-carrier HC. The supplemented dose of 1.5 µg per day is insufficient to reach a fast replenishment of B12 stores and cause a metabolic response in a population with low B12 status.

## Figures and Tables

**Table 1:** Measurements of biomarkers at baseline for individuals destined for intervention with B12 present in capsules, cow milk or buffalo milk.

Marker in Plasma	Reference Range	Capsule Median (min/max)	Cow Milk Median (min/max)	Buffalo Milk Median (min/max)	Kruskal-Wallis, *p* ^1^
*n*, gender		22 = 15M + 7F	23 = 7M + 16F	22 = 8M + 14F	
age (year)		29(21/50)	32(21/55)	33(23/52)	0.28
B12 (pmol/L)	148–600	110 (63/199)	140 (56/258)	111 (51/314)	0.59
Hcy (µmol/L)	5–15	34 (14/106)	22 (11/83)	23 (10/118)	0.05
HoloTC (pmol/L)	40–150	20 (7/94)	45 (10/88)	30 (14/119)	**0.003**
MMA (µmol/L)	0.1–0.3	0.60 (0.15/1.8)	0.53 (0.22/2.2)	0.63 (0.25/1.8)	0.94
cB12 index	(−0.5)–(+1.5)	−2.0 (−3.2/+0.2)	−1.1 (−2.6/−0.2)	−1.5 (−3.1/+0.2)	0.09
folate (nmol/L)	4.5–45	10.4 (7.0/23)	8.2 (4.8/25)	10.2 (3.9/26)	0.11
cB12 index ^2^ (+folate)	(−0.5)–(+1.5)	−1.9 (−3.1/+0.3)	−1.0 (−2.5/−0.1)	−1.4 (−2.9/+0.3)	0.06
Cholesterol (mg/dL)	150–200	172 (107/245)	175 (107/245)	172 (109/204)	0.67
HDL-Cholesterol (mg/dL)	35–55	44 (30/84)	44 (30/84)	42 (28/88)	0.62
Triglycerides (mg/dL)	30–160	86 (43/410)	86 (43/410)	84 (36/231)	0.48

^1^ Probability of the overall identity between the three supplementation groups according to a Kruskal-Wallis test. The underlined and bold figures highlight the increasing levels of significance. ^2^ Combined index cB12 with correction for low folate. F: female; Hcy: homocysteine; HoloTC: holotranscobalamin; M: male; MMA: methylmalonic acid; HDL: high density lipoprotein.

**Table 2:** End-point measurements of B12 related biomarkers after intervention with B12 present in capsules, cow milk, or buffalo milk and their comparison to baselines ^1^.

Markers in Plasma	Capsule Median (min/max);	Cow Milk Median (min/max);	Buffalo Milk Median (min/max);
*n* =	22	23	22
Vitamin B12 (pmol/L)	178 (75/280) ***p* < 0.001**	155 (61/292) ***p* < 0.001**	145 (55/350) ***p* = 0.001**
Hcy (µmol/L)	31 (10/78) ***p* < 0.001**	17 (10/62) ***p* < 0.001**	18 (8/107) ***p* < 0.001**
HoloTC (pmol/L)	27 (8/55) *p* = 0.18	43 (16/92) *p* = 0.39	27 (16/79) *p* = 0.52
MMA (µmol/L)	0.58 (0.13/1.55) *p* = 0.03	0.49 (0.23/1.55) *p* = 0.77	0.57 (0.20/1.90) *p* = 0.64
cB12 index	−1.3 (−2.6/+0.2) ***p* < 0.001**	−0.7 (−2.3/+0.0) ***p* = 0.002**	−1.2 (−2.7/+0.4) ***p* = 0.002**

^1^ Probability of the pairwise identity (baseline vs 3.5 week, see smoothing in Section 2.6) according to a Wilcoxon signed rank test. The underlined and bold figures highlight the increasing levels of significance.

## Supplementary Materials

The following are available online at http://www.mdpi.com/2072-6643/11/2/304/s1, Table S1: Blood collection and B12 measurement in milk; Table S2: Vitamin B12 concentration in milk (uncorrected); Table S3: Fitting parameters of the markers, responding to the treatment with CN-B12 capsules, cow milk or buffalo milk; Figure S1: Dispersion of MMA responses in the three supplementation groups.
