# Supplementary material for: Comparative Bioavailability of Synthetic B12 and Dietary Vitamin B12 Present in Cow and Buffalo Milk: A Prospective Study in Lactovegetarian Indians
Source: Nutrients. 2019 Feb 1;11(2):304. doi: 10.3390/nu11020304 (PMC6412291; doi:10.3390/nu11020304)
Supplement: Supplementary file 1 [file nutrients-11-00304-s001.pdf]

Supplementary Table S1. Blood collection and B12 measurement in milk

**Time of blood collection**	**Investigations**	**Vitamin B12 measurement in milk [8]**
Day-0	Complete blood count, lipids, creatinine, vitamin B12, holotranscobalamin, homocysteine, MMA	Cow & buffalo
1st week	vitamin B12, holotranscobalamin, homocysteine, MMA	
2nd week	vitamin B12, holotranscobalamin, homocysteine, MMA	Cow & buffalo
3rd week	vitamin B12, holotranscobalamin, homocysteine, MMA	
4th week	Haemogram, lipids, creatinine, vitamin B12, holotranscobalamin, homocysteine, MMA	

Supplementary Table S2. Vitamin B12 concentration in milk (uncorrected) [a]

**Measured on day**	**Vitamin B12 (µg/L) [a] in milk from M/S Chitale Bandhu dairy [8]**	
	Cow	Buffalo
-14	3.05	3.25
-13	2.85	3.10
-12	3.20	3.00
start 0	2.90	3.15
14	3.20	3.00

[a] Measurements of B12 in milk samples were performed according to the standard procedures of Architect (Abbott) and ADVIA Centaur XP (Siemens). A separate follow up assay indicated that a preliminary extraction of milk samples increased the measured B12 by factor ≈ 1.3 (sample or standard +1 mM KCN, 100 ºC, 20 min; +2% SDS, 100 ºC, 20 min; +0.2 M KCl, 0 ºC, 20 min; supernatant used). Therefore, the results from Table S2 were multiplied by 1.3, giving the final value of B12 ≈ 3.9 µg/L (0.78 µg/200 mL) in both samples.

Supplementary Table S3. Fitting parameters of the markers, responding to the treatment with CN-B12 capsules, cow milk or buffalo milk.

\ Marker Fit\	**CN-B12 fitting P_i**	**probability of ref. value**	**cow milk fitting P_i**	**probability of ref. value**	**buffalo milk fitting P_i**	**probability of ref. value**
ΔB12	Eq. 1 (no P_3)		Eq. 1 (no P_3)		Eq. 1 (no P_3)	
$P_1 \pm$ SE	not	not	0.0 ± 4.8	1.0	0.0 ± 4.5	1.0
$P_2 \pm$ SE	applicable	applicable	10.5 ± 2.1	$3 \cdot 10^{-6}$	10.3 ± 2.0	$1 \cdot 10^{-6}$
(p overall)				**($3 \cdot 10^{-6}$)**		**($1 \cdot 10^{-6}$)**
ΔB12	Eq. 2		Eq. 2		Eq. 2	
$P_1 \pm$ SE	0.0 ± 6.3	1.0	0.0 ± 5.5	1.0	0.0 ± 5.0	1.0
$P_2 \pm$ SE	–14.8 ± 9.8	0.13	10.3 ± 8.6	0.24	3.7 ± 7.9	0.64
$P_3 \pm$ SE	49.8 ± 17.7	0.006	0.42 ± 15.6	0.98	11.1 ± 14.3	0.44
(p overall)		**($8 \cdot 10^{-4}$)**		(0.24)		(0.28)
ΔholoTC	Eq. 2		Eq. 2		Eq. 2	
$P_1 \pm$ SE	0.0 ± 2.0	1.0	0.0 ± 2.6	1.0	0.0 ± 2.0	1.0
$P_2 \pm$ SE	–5.2 ± 3.2	0.12	–10.7 ± 4.1	0.01	–11.6 ± 3.2	$5 \cdot 10^{-4}$
$P_3 \pm$ SE	10.6 ± 5.8	0.07	21.5 ± 7.4	0.005	22.7 ± 5.7	$2 \cdot 10^{-4}$
(p overall)		**(0.008)**		**($5 \cdot 10^{-5}$)**		**($1 \cdot 10^{-7}$)**
R(MMA)	Eq. 1		Eq. 1		Eq. 1	
$P_1 \pm$ SE	1.00 ± 0.03	1.0	1.00 ± 0.07	1.0	1.00 ± 0.07	1.0
$P_2 \pm$ SE	–0.11 ± 0.04	0.01	0.015 ±	0.65	0.001 ± 0.09	0.99
$P_3 \pm$ SE	0.019 ± 0.01	0.11	0.031	0.71	0.003 ± 0.03	0.91
(p, overall)		**(0.001)**		(0.46)		(0.90)
R(Hcy)	Eq. 3		Eq. 3		Eq. 3	
$P_1 \pm$ SE	1.00 ± 0.02	1.0	1.00 ± 0.02	1.0	1.00 ± 0.02	1.0
$P_2 \pm$ SE	–0.26 ± 0.06	$6 \cdot 10^{-5}$	–0.25 ± 0.03	$6 \cdot 10^{-12}$	–0.21 ± 0.04	$1 \cdot 10^{-7}$
$P_3 \pm$ SE	0.60 ± 0.33	0.08	1.15 ± 0.51	0.03	1.07 ± 0.62	0.09
(p, overall)		**($5 \cdot 10^{-6}$)**		**($2 \cdot 10^{-13}$)**		**($1 \cdot 10^{-8}$)**

The underlined and bold figures highlight the increasing levels of significance for deviation from a "zero response".
